# Application of Response Surface Methodology to Evaluate Photodynamic Inactivation Mediated by Eosin Y and 530 nm LED against *Staphylococcus aureus*

**DOI:** 10.3390/antibiotics9030125

**Published:** 2020-03-17

**Authors:** Adriele R. Santos, Alex F. da Silva, Andréia F. P. Batista, Camila F. Freitas, Evandro Bona, Maria J. Sereia, Wilker Caetano, Noburu Hioka, Jane M. G. Mikcha

**Affiliations:** 1 Postgraduate Program in Food Science, State University of Maringá, Maringá 87020-900—Paraná, Brazil; andreia.farias04@hotmail.com; 2 Postgraduate Program in Health Science, State University of Maringá, Maringá 87020-900—Paraná, Brazil; alexfiorisilva@gmail.com; 3 Department of Chemistry, State University of Maringá, Maringá 87020-900—Paraná, Brazil; camila.freitas1989@hotmail.com (C.F.F.); wcaetano@uem.br (W.C.); nhioka@uem.br (N.H.); 4 Department of Food, Federal Technological University of Paraná, Campo Mourão 87301-899—Paraná, Brazil; ebona@utfpr.edu.br (E.B.);; 5 Department of Clinical Analysis and Biomedicine, State University of Maringá, Maringá 87020-900—Paraná, Brazil

**Keywords:** photodynamic inactivation, mathematical model, xanthene dye, foodborne pathogen, green LED light

## Abstract

Photodynamic antimicrobial chemotherapy (PAC) is an efficient tool for inactivating microorganisms. This technique is a good approach to inactivate the foodborne microorganisms, which are responsible for one of the major public health concerns worldwide—the foodborne diseases. In this work, response surface methodology (RSM) was used to evaluate the interaction of Eosin Y (EOS) concentration and irradiation time on *Staphylococcus aureus* counts and a sequence of designed experiments to model the combined effect of each factor on the response. A second-order polynomial empirical model was developed to describe the relationship between EOS concentration and irradiation time. The results showed that the derived model could predict the combined influences of these factors on *S. aureus* counts. The agreement between predictions and experimental observations (R^2^_adj_ = 0.9159, *p* = 0.000034) was also observed. The significant terms in the model were the linear negative effect of photosensitizer (PS) concentration, followed by the linear negative effect of irradiation time, and the quadratic negative effect of PS concentration. The highest reductions in *S. aureus* counts were observed when applying a light dose of 9.98 J/cm^2^ (498 nM of EOS and 10 min. irradiation). The ability of the evaluated model to predict the photoinactivation of *S. aureus* was successfully validated. Therefore, the use of RSM combined with PAC is a promising approach to inactivate foodborne pathogens.

## 1. Introduction

Foodborne diseases are a public health problem that compromise health care systems and harm national economies; besides this, they are an important cause of morbidity and mortality worldwide [1]. According to the Centers for Disease Control and Prevention [2], *Staphylococcus aureus* was responsible for 671 outbreaks with 526 hospitalizations between 1998–2016 in the United States, becoming one of the top five pathogens that cause foodborne disease.

Preventing outbreaks of foodborne disease requires the control of microorganisms in the food production chain. However, the conventional methodologies used in food preservation are related to some undesirable characteristics such as the possibility of induced physical and chemical changes in food, high costs, and a requirement for high investment and specialized equipment [3,4]. In this context, an efficient tool for inactivating microorganisms is photodynamic antimicrobial chemotherapy (PAC), which is a promising and low-price technology that is effective against a several types of foodborne bacteria [3,5,6,7,8]. In PAC, a light-excited photosensitizer (PS), in the presence of molecular oxygen, produces reactive oxygen species (ROS), such as singlet oxygen and/or hydroxyl radicals, superoxide, and hydrogen peroxide [9,10,11,12,13]. 

The mechanism of action of PAC consists of electron transfer reactions or energy transfer from the light to the surrounding oxygen (O_2_) [4]. The PS, in presence of an appropriate wavelength light, absorbs energy forming the very unstable excited-state PS [14]. This state loses its excess energy and, by fluorescence emission, can return to the singlet state or can be converted to a stable and longer lifetime excited triplet state [4,14,15]. The excited triplet state PS can react with oxygen via two pathways (type I and type II). The type I photochemical process occurs when electron transfer reactions of excited state PS to molecular oxygen form reactive oxygen species [4,14,15]. In type II pathway the triplet state PS can directly transfer energy to molecular oxygen, by colliding with it and leading to the formation of singlet oxygen (^1^O_2_) [4,14,15]. These reactions promote the inactivation of microorganisms by irreversible damage to various molecular constituents of cells (lipids, proteins, enzymes, and DNA) [16]. 

The efficiency of PAC depends on the chemical structure of the PS used [15]. One of the essential requirements of a PS is to have a high ^1^O_2_ quantum yield (Φ∆), however, an ideal PS should have other characteristics such as high absorption coefficient, photostability, solubility, capacity of absorbing, and using energy to excite oxygen to its singlet state, selectivity for the target cells, have no dark toxicity and high quantum yield of triplet state [15,17,18]. Several compounds have been studied as PS to be used in PAC, namely phenothiazine derivatives, xanthene dyes, chlorophyllins, porphyrins, and phthalocyanines. [4,6,12,13,14]. Among these compounds, the xanthene dyes are an inexpensive PS, show low toxicity in the dark, high singlet oxygen quantum yield, and are effective to control the foodborne bacteria [3,6,14,17,19,20,21]. Eosin (EOS), a xanthene dye, is highly soluble in water at physiological pH, has high molar absorptivity, high formation of singlet oxygen quantum yield (0.57), and high light absorption in the visible region (ε (517 nm) = 96.600 mol cm/L), which make it a good PS to induce bacterial photoinactivation [3,14,17]. In addition, this dye is approved for use in drugs and cosmetics as a color additive [3].

To reach the antimicrobial effects of PAC, the interactions of oxygen, PS and light source are crucial [15]. The PS and light sources have distinct absorption and emission characteristics. So, the radiation provided by the light source must be in the region of the maximum absorption wavelength of the dye [15]. Nowadays, there are several light sources available (incandescent lamps, arc xenon lamps, metal halide lamps, and fluorescent lamps) that provide a broad range of wavelengths [15,22]. One of the most interesting is the light-emitting diode (LED), as it has significant advantages for clinical, food, and laboratory uses [18,22]. The nonexistence of hazardous agents (heavy metals), resistance to shock and vibration, and a wide wavelength emission range are some of them [15,18,22]. This light system is also versatile (can be arranged to irradiate large areas) and cheaper than other light sources commonly used in PAC [15,22].

In practice, to perform the PAC assays, it is necessary to consider three variables: PS concentration, dark incubation time, and irradiation time [23]. One method to test all the variable combinations is a step-by-step approach, which means, each factor is varied while the other ones are fixed to an arbitrary value [23,24]. However, this method requires a large number of experiments and would provide biased results, once there are some interactions between factors [23]. In this sense, response surface methodology (RSM), a method that combines statistical and mathematical techniques to infer a multivariate model, could optimize the analyses significantly reducing the number of experiment trails [23,24,25]. This method uses quantitative data from designed experiments and could define the relationships between the response and independent variables [23,24,25]. RSM has already been successfully applied in various fields and could be a valuable tool for applying PAC in the food industry [23]. Moreover, the RSM is an alternative and empirical approach to optimize the investigation of new PS. 

So, the present study was undertaken to develop response surface models to evaluate the interaction of eosin Y concentration and illumination time using green light-emitting diodes in the PAC against *Staphylococcus aureus* ATCC 25923.

## 2. Results and Discussion 

### 2.1. Light Doses

In this study, the values of light doses (Table 1) were calculated using Equations (1) and (2), which consider the light power emitted by LED (P_Led emit_) and absorbed by PS (P_Abs_). Furthermore, Gerola et al. [26] also consider the PS photobleaching reaction and spectral overlap between the light source and the PS in their calculations, which is rare in most studies that evaluate PAC effectiveness. Therefore, it is expected that the light doses obtained in this study differ, not only for different irradiation times but also for different PS concentrations.

**Figure 1:**
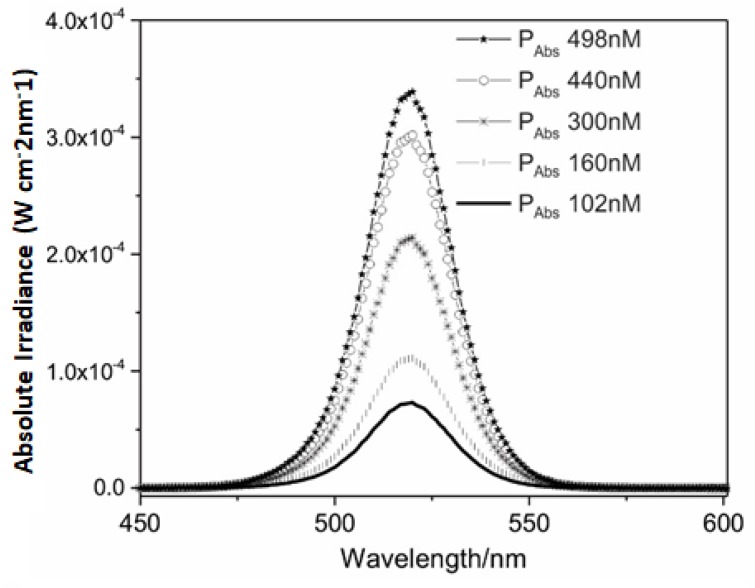
Spectra of light emitted by light-emitting diode (LED) (P_LED Emitted_) and power absorbed by eosin (P_Abs_) in different concentrations (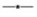 498 nM; 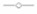 440 nM; 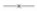 300 nM; 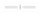 160 nM; and 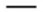 102 nM).

**Figure 2:**
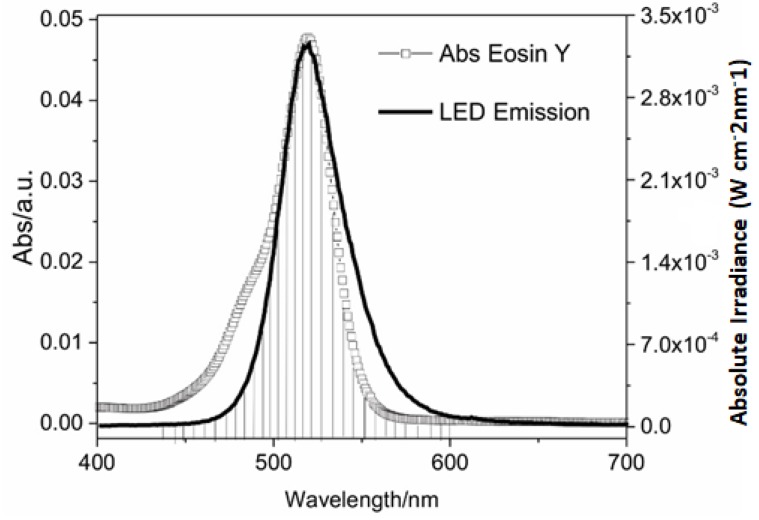
Light-emitting diode emitted potency (P_LED Emitted_) and absorbed potency by eosin Y (P_Abs_).

It is important to note that the maximum power absorbed (P_Abs_) by EOS was around 3.5 × 10^−4^ W cm^−2^ nm^−1^ (Figure 1), while the power emitted by LED (P_Led emit_) was around 3.5 × 10^−3^ W cm^−2^ nm^−1^ (Figure 2). This suggested that only a small part of the light emitted by LED was absorbed by the PS. So, the light doses methodology proposed by Gerola et al. [26] is relevant because it allows measurement of the exact fraction of LED power that was absorbed by the different concentrations of PS and lighting times.

The spectral absorption profile of EOS is quite similar to the spectral profile of LED emission (Figure 2). To reach better results with PAC is necessary a good combination of PS absorption and light emission wavelengths [20]. This is clearly observed with the EOS and the green LED light, where the hatched area in Figure 1 shows the power emitted by the light source that is effectively absorbed by the PS. It is noteworthy that the use of green LEDs (wavelength of 530 ± 40 nm) provides a large overlap between the absorbance of EOS and the emission of LED lights. So, the power absorbed is greater when using an LED lamp with this wavelength, compared to other kinds of light, improving the efficiency of the PAC. Therefore, the green LED light used in this study is adequate for EOS activation.

### 2.2. Photodynamic Inactivation of Staphylococcus aureus

Photodynamic antimicrobial chemotherapy is a valuable technology to inactivated foodborne pathogens, to extend the shelf-life of food and to be used as a sanitizer in the food industry [3,27]. Moreover, this technology does not form toxic products and nor leads to the development of microbial resistance [15,21]. The present study showed that the combination of EOS and green LED has a great effect on *S. aureus* cell viability.

For the controls tested, no significant difference (*p* < 0.05) in bacterial counts was observed. The PS−L− control showed counts of 6.10 ± 0.05 log CFU/mL, while PS+L− and PS−L+ showed 6.00 ± 0.056 log CFU/mL and 6.10 ± 0.06 log CFU/mL, respectively. 

In this study, the highest reduction in counts was approximately 2 logs CFU/mL when the bacterial strain was treated with a concentration of 498 nM of PS with an irradiation time of 10 min, which is equivalent to a light dose of 9.98 J/cm^2^ (Table 1). Johnson et al. [28], comparing the effect of EOS and peptide-conjugated (KLAKLAK)2 EOS on *S. aureus*, reported no effect on cell viability when using a concentration of 10 μM of PS and 30 min of irradiation with a halogen light with a green filter lamp. Kato et al. [19], working with different xanthene dyes, showed that eosin B at 5 μM and 10 min irradiation with a halogen lamp was not able to reduce the *S. aureus* counts.

In this study, even working with lower PS concentrations and irradiation time than those in the mentioned studies, we achieved better results in the photoinactivation of *S. aureus*. These results can be explained by the kind of light used in our research, since xanthene dyes absorb light within a range that corresponds to the light emitted by green LEDs [20].

### 2.3. Determination of Photoinhibitory Activity of Eosin Y and Green LED Light Using the Statistical Experimental Design 

The results in Table S1 (ANOVA) demonstrated that this mathematical model is highly significant (*p* = 0.000034) and did not show lack of fit (*p* = 0.401258). The R^2^_adj_ value of 0.9159 indicates that 91.59% of the total variation is explained by the model. Considering these results and the natural variation of the microbiological experimentation [29], this mathematical model is adequate for predicting the photo inhibitory activity of EOS with green LED light against *S. aureus.*

**Figure 3:**
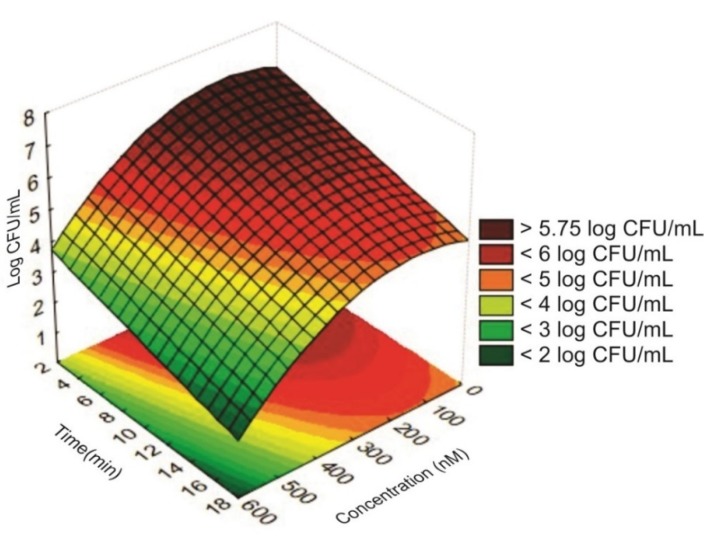
Response surface describing interactive influence of the photosensitizer (PS) concentration and irradiation time on *S. aureus* counts.

As only significant coefficients (*p* < 0.05) were considered (Table S2—Supplementary Material) in the mathematical models, the interactions between PS concentration and irradiation time and the quadratic effect for the variable irradiation time (Table S2) were not used to compose the model. The linear effect of PS concentration was the most significant term (*p* = 0.002294) in the model, followed by the linear effect of irradiation time (*p* = 0.006219) (Table S3—Supplementary Material). This means that the PS concentration has a greater influence than the irradiation time in reducing the counts of *S. aureus.* Bonin et al. [3] observed total inactivation on *S. aureus* counts using a shorter time (5 min) and higher PS concentration (5 µM), corroborating data obtained by the RSM. Moreover, the ANOVA showed that these variables exhibited negative effects (Table S2 and Figure 3), in other words, the bacterial counts tend to decrease as the PS concentration or the irradiation time increase. This indicates that the photo inhibitory effect is lower until approximately 100 nM (Figure 3). However, when EOS concentration is higher than 200 nM, the photo inhibitory effect is accentuated (Figure 3).

Today, the application of RSM in the optimization of analytical procedures is largely diffused and consolidated because of its several advantages compared to the classical optimization methods, in which a one-variable-at-a-time technique is used [21,24,25]. RSM can thus allow the prediction of the PS concentration and irradiation time required to reach a certain bacterial inhibition with a small number of experiments, which reduces time consumption and cost of the research. 

### 2.4. Model Validation 

One of the critical steps in the development of mathematics models is its validation. [30]. The ability of our model to predict the photoinactivation of *S. aureus* was validated against data not used in its development, collected using the aforementioned criteria. So, to evaluate the data acquired for model development and validation, it was necessary to compare the observed and predicted values (Table 2) using a 95% confidence interval. As shown in Table 2, the three experiments tested are within the 95% confidence interval. Despite the third experiment has a value of 4.08 logs CFU/mL, it is within the 95% confidence interval because of its standard deviation of ± 0.28 (4.08 − 0.28 = 3.80). Thus, the model was successfully validated because it provided acceptable predictions for the data for interpolation.

## 3. Materials and Methods 

### 3.1. Bacterial Strains and Culture Conditions

In this study, *Staphylococcus aureus* ATCC 25923 strain, stored at −20 °C in a 20% (vol/vol) glycerol Brain and Heart Infusion Broth, was used (Difco, Becton Dickinson, Sparks, MD, USA).

In all experiments, the inoculum prepared according to Santos et al. [21] was used. The bacterium was grown overnight at 37 °C, in Tryptic Soy Broth (Difco), harvested by centrifugation at 5000× *g* for 4 min, and washed three times and resuspended in sterile phosphate-buffered saline (PBS; pH 7.2). The inoculum was then standardized in a spectrophotometer at 580 nm (%T 25–30) to produce a bacterial suspension containing approximately 10^8^ colony-forming units (CFU)/mL. This standardized suspension was diluted in PBS to approximately 10^7^ CFU/mL for use in the experiments.

### 3.2. Photosensitizers and LED Light Source

A stock solution of EOS (Sigma, St. Louis, MO) was prepared in PBS with a concentration of 1 × 10^−3^ M. This stock solution was sterilized by filtration (0.22 µm) and standardized in a spectrophotometer (UV-Vis Beckman Coulter DU *800) at wavelength λ max = 518 nm [21].

The LED device used in the experiments is a homemade prototype (13 cm length by 8 cm width; 3.5 cm distance from the sample surface) with a fluency rate of 10 mW/cm^2^ and a maximum emission wavelength (λemiss) of 530 ± 40 nm. To determine the absolute irradiance of the LED a Spectroradiometer USB2000 + RAD (Ocean Optics, Winter Park, FL) was used. To obtain the spectral emission of the LED system was used a spectrofluorometer (Varian Cary Eclipse, San Diego, CA). To calculate the LED beam array and the light doses (D_Abs_) was used the methodology described by Gerola et al. [26] (Equations (1) and (2)).

(1) $$D_{\mathrm{Abs}}=\frac{t}{A}\;\cdot \;\int_{\lambda 2}^{\lambda 1}P_{\mathrm{Abs}}\;\delta \lambda \;$$

A is the irradiated area; t is the exposure time, and P_Abs_ was calculated according to the following equation:

(2) $$P_{Abs}=X_{Abs}\;.\;P_{LED\;emited}$$

X_Abs_ is the absorbed light fraction by the PS, P_Abs_ is the absorbed potency by the PS, and P_LED emitted_ is the LED potency.

### 3.3. Photodynamic Inactivation of Staphylococcus aureus

An aliquot of 25 µL of bacterial suspension standardized at 10^7^ CFU/mL was homogenized with 475 µL EOS at different concentrations in a 24-well microplate; the mixture was incubated for 10 min in the dark, and then illuminated with a green LED light (PS+L+) up to the maximum time of 15.65 min (Table 1). The PS control (bacterium and PS without irradiation—PS+L−), the light control (bacterium in PBS under irradiation—PS−L+), and the positive control (bacterium in PBS without irradiation—PS−L−) were also evaluated [21]. 

After the PAC treatment, 100 µL of the samples and controls were serially diluted in 0.85% saline solution. To determine the CFU/ml, 10 µL of each dilution were plated on Trypticase Soy Agar (Difco) plates. The plates were incubated 37 °C for 24 h [21].

### 3.4. Determination of Photoinhibitory Activity of Eosin Y Using the Statistical Experimental Design 

To establish the conditions for the photoinhibitory activity of EOS against *S. aureus,* a Rotational Central Composite Design (Table 1), generated by the software Statistica 13 (TIBCO Software, Inc., 2017), was used. There were eight experiments with four repetitions of the central point performed to evaluate the combined effects of two independent variables: The concentrations of EOS (100 to 500 nM)—X1 and the irradiation time (5 to 15 min)—X2. 

### 3.5. Statistical Analysis

The bacterial counts were carried out in six replicates, and the results were presented as means ± standard deviations. A second-order polynomial model was used for fitting the experimental data, and its coefficients were obtained by multiple linear regressions (Equation (3)).

(3) $$Ŷ=b_{0}+b_{1}X_{1}+b_{2}X_{2}+b_{11}X_{1}^{2}+b_{22}X_{2}^{2}+b_{12}X_{1}X_{2}$$

where Ŷ is the predicted response; b_0_ is the regression coefficient for the intercept; b_1_ and b_2_ are the regression coefficients representing the linear effect terms; b_11_ and b_22_ are the quadratic effect terms; and b_12_ is the interaction effect terms and X_1_ and X_2_ are the independent variables in coded values. 

A t-test was used to analyze the regression coefficients of the mathematical models and excluded those that were not significant (*p* > 0.05). To determine the significance of the model, the ANOVA (*p* < 0.05), the coefficients of determination (R^2^) and the adjusted coefficients of determination (R^2^_adj_) were used. The Pearson coefficient was used for estimating the correlation among the original experimental responses. The software Statistica 13 (TIBCO Software, Inc., 2017) was used for the data statistical treatment described above and for generating the response surface plots.

### 3.6. Model Validation

The model was validated using the limit values of the independent variables determined by rotational central composite design. Three combinations were used: (1) highest concentration with shortest time (498 nM and 4.34 min); (2) lowest concentration with longest time (102 nM and 15.65 min); and (3) highest concentration with highest time (498 nM and 15.65 min). 

The experimental results (mean ± standard deviation) were compared with the predicted values of the mathematical models under the experimental conditions one, two, and three, considering the 95% confidence interval. 

## 4. Conclusions

In sum, the polynomial model developed in the present work was able to provide accurate information on the combined influence of EOS concentration and irradiation time in PAC, to predict the photo inhibitory activity against *S. aureus*. According to the RSM results, the PS concentration was the variable that most influenced the PAC. So, this is an important variable to be consider in future research, to achieve the complete inactivation of *S. aureus*. Using the experimental design, it is possible to perform analyses in which all parameters can be varied at the same time, decreasing the number of tests needed, costs, and the time spent to achieve the results. In this work, the mathematical model was developed only with *S. aureus*; however, it could be done with another genus of bacteria (e.g., Gram negative) in future studies. Therefore, the use of RSM combined with PAC is a promising approach to the inactivation of foodborne pathogens.

## Figures and Tables

**Table 1:** Experimental design (coded and real values) used to determine the combined influence of two independent variables (eosin (EOS) concentration and irradiation time) on *S. aureus* cells viability. The EOS concentration and irradiation time was used to calculate the light dose, according to Gerola et al. [26].

Experiments	Coded Values		Real Values			
	X1	X2	Concentration (nM)	Time (min)	Light Doses (J/cm^2^ [15])	Cell Viability (Log CFU/mL) **
Control (PS−L−) *	--	--	0	0	0	6.23 ± 0.06
1	−1.00000	−1.00000	160	6.00	1.96	6.24 ± 0.07
2	−1.00000	1.00000	160	14.00	4.56	5.17 ± 0.02
3	1.00000	−1.00000	440	6.00	5.32	5.17 ± 0.06
4	1.00000	1.00000	440	14.00	12.43	4.14 ± 0.03
5	−1.41421	0.00000	102	10.00	2.13	6.20 ± 0.02
6	1.41421	0.00000	498	10.00	9.98	3.91 ± 0.02
7	0.00000	−1.41421	300	4.34	2.72	6.30 ± 0.07
8	0.00000	1.41421	300	15.65	9.84	5.11 ± 0.05
9	0.00000	0.00000	300	10.00	6.30	5.49 ± 0.01
10	0.00000	0.00000	300	10.00	6.30	5.31 ± 0.02
11	0.00000	0.00000	300	10.00	6.30	5.53 ± 0.06
12	0.00000	0.00000	300	10.00	6.30	5.78 ± 0.04

* Positive control, containing only the inoculum in PBS and without irradiation. ** Values are mean followed by standard deviation.

**Table 2:** Comparison between predicted and observed values for cell viability of the three experiments tested for validation of the regression models.

Responses	Cell Viability (Log CFU/mL)		
	Experiment 1	Experiment 2	Experiment 3
Predicted	4.70	5.25	3.36
PCILL-95% ^a^	4.23	4.78	2.89
PCIUL-95% ^b^	5.16	5.71	3.82
Observed	5.13 ± 0.11	5.09 ± 0.24	4.08 ± 0.28

^a^ Lower limit of the predicted confidence interval at 95%.; ^b^ Upper limit of the predicted confidence interval at 95%.

## Supplementary Materials

The following are available online at https://www.mdpi.com/2079-6382/9/3/125/s1, Table S1: Analysis of variance for the evaluation of the second-order polynomial model, Table S2: Regression coefficients of the mathematical model to predict the photoinhibitory effects of eosin Y and green LED light against *S. aureus*. Table S3: Analysis of variance for the significant terms in the model.
