# Supplementary material for: Application of Response Surface Methodology to Evaluate Photodynamic Inactivation Mediated by Eosin Y and 530 nm LED against Staphylococcus aureus
Source: Antibiotics (Basel). 2020 Mar 17;9(3):125. doi: 10.3390/antibiotics9030125 (PMC7148482; doi:10.3390/antibiotics9030125)
Supplement: Supplementary file 1 [file antibiotics-09-00125-s001.pdf]

SUPLEMENTARY MATERIAL

Application of response surface methodology to evaluate photodynamic inactivation mediated by eosin Y and 530 nm LED against *Staphylococcus aureus*.

Adriele Rodrigues dos Santos*, Alex Fiori da Silva, Edineia Bonin, Andréia Farias Pereira Batista, Camila Fabiano de Freitas, Evandro Bona, Maria Josiane Sereia, Wilker Caetano, Noboru Hioka, Jane Martha Graton Mikcha*.

*Corresponding Author: Laboratório de microbiologia de alimentos, Departamento de análises clínicas e biomedicina, Universidade Estadual de Maringá, Avenida Colombo, 5790, zona 07, Maringá, 87020-900, PR, Brasil.

e-mail: adrielesantos@hotmail.com; jmgmikcha@uem.br

Table S1. Analysis of variance for the evaluation of the second-order polynomial model.

**Source of variation**	**Sum of squares**	***df***	**Mean square**	***F*-ratio**	***p*-value**
Model	5.9276	3	1.9759	40.92	0.000034
Residual	0.3863	8	0.0483		
Lack of fit	0.2738	5	0.0548	1.46055	0.401258
Pure error	0.1125	3	0.0375		
Total	6.3138	11			

Coefficient of determination (R^2) = 0.93882;

Adjusted coefficient of determination (R^2_{adj}) = 0.91588

Table S2. Regression coefficients of the mathematical model to predict the photoinhibitory effects of eosin Y and green LED light against *S. aureus.*

**Independent variables***	**Regression coefficient**	**Standard error**	***t*-value**	***p*-value**
X_1	-0.667319	0.088536	-7.53723	0.000283
X_1^2	-0.286250	0.098987	-2.89180	0.027628
X_2	-0.472864	0.088536	-5.34091	0.001760
X_2^2	0.038750	0.098987	0.39147	0.708977
X_1X_2	0.020000	0.250419	0.07987	0.938941

*X_1 – linear effect of PS concentration; X_2 – linear effect of illumination time; X_1^2 – quadratic effect of PS concentration; X_2^2 – quadratic effect of illumination time; X_1X_2 - interaction between PS concentration and illumination time.

Table S3. Analysis of variance for the significant terms in the model

**Independent variables***	**Sum of squares**	***df***	**Mean square**	***F*-ratio**	***p*-value**
X_1	3.562513	1	3.562513	95.02147	0.002294
X_1^2	0.576240	1	0.576240	15.36982	0.029517
X_2	1.788805	1	1.788805	47.71207	0.006219

*X_1 – linear effect of PS concentration; X_2 – linear effect of illumination time; X_1^2 – quadratic effect of PS concentration; X_2^2.
